# Sulphurous Vapor Baths in Chronic Rheumatism

**Published:** 1860-03

**Authors:** G. L. Purdy


*Sulphurous Vapor Baths in Chronic Rheumatism.* By G. L. Purdy, M. D.

[From the Cleveland Medical Gazette.]

All practitioners of much experience, are aware of the obstinancy of this affection—chronic Rheumatism, how slow it is to yield to remedies, and how often it entirely defies the whole array of Therapeutics. And under these circumstances, any means that tend towards its alleviation or cure ought to be promulgated among the profession, and used for the benefit of sufferers from this disease.

The treatment of chronic rheumatism by the sulphurous vapor baths, is reported by James Williams, M. D., in the *London Lancet* for August, 1858, and in the thirty-eighth number of *Braithwaite's Retrospect*, the same thing is upon record.

I shall not attempt to enter into the pathalogy of this disease, nor the *modus operandi* of the treatment, but will merely make up a short report of a case in which I used the bath as an adjuvant to other means, with a successful result, after the disease had resisted similar means, unaided by the bath.

Dr. Williams describes an apparatus for the administration of the bath, but it can be used without going to all of this trouble.

I had my patient stripped to the skin, and seated on a tight bottomed chair—thickly enveloped with flannel blankets from the shoulders downwards. Place the blankets tightly around the neck, and have them fit snugly to the floor, around the patient's chair—but as far from it as you conveniently can, so there will be no danger of their taking fire from the flame of the sulphur. Now, place under the patient's chair an iron vessel containing one fourth of an ounce of sublimed sulphur of the shops, and upon the sulphur, place a small piece of red hot iron, and combustion immediately takes place. If the vapor should escape through the blankets too much, and thus annoy the patient, place more covering around him, or keep the vapor from the face with a fan.

The patient should be thus vaporized 20 or 30 minutes, or until a very free perspiration is got up. If the sulphur burns out too soon, add some more. When the bath is discontinued have the blankets removed, and the patient well sprinkled with a half gallon of cold salt water, and thoroughly dried by brisk friction, and placed in bed, warmly covered for three hours. The bath should be used once every second or third day.

Mrs. Y., aged about 30, was attacked last March, with rheumetism of the left hip, knee and ankle joints, incapacitating her from going about, except by the aid of crutches—sometimes she could not even use these for days at a time.

From what I could learn, the disease was rather of the acute character at its commencement. She was treated for six weeks by a medical friend of a neighboring village, who subjected her to all the remedies usually applied in such cases, but with very little benefit.

She then underwent a two months' course of infinitesimal homœopathy, but received no beneficial results. The homœopathist promised a cure in two weeks.

From this time, until the 12th of last August, she went the rounds of home-made and patent medicines, the results as before—no benefit of a permanent character.

I was called to visit her for the first time, on the 12th of last August, and found her almost helpless from inability to use the left leg; the joints being quite sensitive to the touch, especially the hip joint. She was very much exhausted, and worn down from loss of sleep, and irritation. Being favorably impressed by Dr. Williams' paper on the "Sulphurous Vapor Bath," I determined to give it a trial in this case. I therefore subjected her to its influence every third day, and gave her 10 grains pulvo. gum guaiac, and 1 grain pulvo. sem cocihicum, three times a day. Under this treatment, and a succession of blisters around the hip joint, she gradually and steadily improved.

By the 25th of September, she could walk without crutches. I now changed the guaiac and colchicum, for the comp. syrup sarsaparilla and iodide potassa. This course was continued for three weeks longer, but the bath was used only once a week since the 25th of September, and discontinued on the 15th of October, when she was well enough to do her own house-work, and discharged her hired girl. She continued the use of the sarsaparilla and iodide of potassa for three weeks longer. As she appeared well, the medicine was discontinued at this time. I frequently hear from her, and her health remains good. I attributed the chief part of her recovery to the sulphurous vapor bath, for in conversation with her first attending physician, he told me that he had given her colchicum, guaiacum, iodide potassa, and many other remedies.

I have given a very condensed account of the case, and yet I think extensive enough, to give an opportunity to see the effects of the bath in this obstinate case of chronic rheumatism.

